# Effect of acupuncture on the opening time of implantation window and endometrial receptivity in controlled ovarian hyperstimulation rats during peri-implantation period

**DOI:** 10.3389/fendo.2023.1110266

**Published:** 2023-03-17

**Authors:** Runan Hu, Yanjing Huang, Yufan Song, Xiao Wu, Kunkun Song, Guangying Huang, Mingmin Zhang, Haoxu Dong

**Affiliations:** ^1^ Institute of Integrated Traditional Chinese and Western Medicine, Tongji Hospital, Tongji Medical College, Huazhong University of Science and Technology, Wuhan, China; ^2^ Department of Integrated Traditional Chinese and Western Medicine, Tongji Hospital, Tongji Medical College, Huazhong University of Science and Technology, Wuhan, Hubei, China

**Keywords:** acupuncture, controlled ovarian hyperstimulation, sex steroid hormones, implantation window, endometrial receptivity

## Abstract

**Purpose:**

To investigate the effect of acupuncture for improving the pregnancy rate of COH rats from the viewpoint of regulating the opening time of the implantation window and endometrial receptivity.

**Methods:**

Experimental rats were randomly divided into normal group (N), model group (M) and acupuncture group(A), and samples were collected on Day 4, 5 and 6 after mating. COH rats were treated with acupuncture at SP6, LR3, and ST36 once a day for 7 times. The pinopodes were observed under a scanning electron microscope. Serum estrogen and progesterone levels were measured *via* ELISA. The protein and mRNA levels of estrogen receptor (ER), progesterone receptor (PR), leukemia inhibitory factor (LIF), integrin β3, vascular endothelial growth factor (VEGF), and fibroblast growth factor 2 (FGF-2) in the endometrium were evaluated *via* West-blot, immunohistochemistry, and PCR.

**Results:**

Compared with group N, the pregnancy rate of group M was significantly decreased (*P*<0.05), and the abnormal serum hormone levels and implantation window advancement were observed. Compared with group M, the pregnancy rate of group A was significantly increased (*P*<0.05), the supraphysiological serum progesterone levels were restored to normalcy (*P*<0.05), and the advanced implantation window was restored to a certain extent. Further, the abnormal ER, PR, LIF, integrin β3, VEGF, and FGF-2 expression levels of the endometrium got recovered to varying degrees.

**Conclusion:**

Acupuncture may restore the estrogen and progesterone balance in COH rats and the forward shift of the implantation window to a certain extent, improving the endometrial receptivity and finally improving the pregnancy rate of COH rats.

## Graphical Abstract

The embryo implantation window moving forward after COH and asynchrony with the development of the endometrium may be important factors associated with a reduced pregnancy rate in COH rats. Acupuncture may restore the estrogen and progesterone balance in COH rats and the forward shift of the implantation window to a certain extent, finally improving the pregnancy rate of COH rats. D, The embryo day; LIF, Leukemia inhibitory factor; Int b3,Integrin b3; VEGF, Vascular endothelial growth factor; FGF-2, Fibroblast growth factor 2; PR, Progesterone receptor; ER, Estrogen receptor; N, Normal group; M, Model group; A, Acupuncture group.

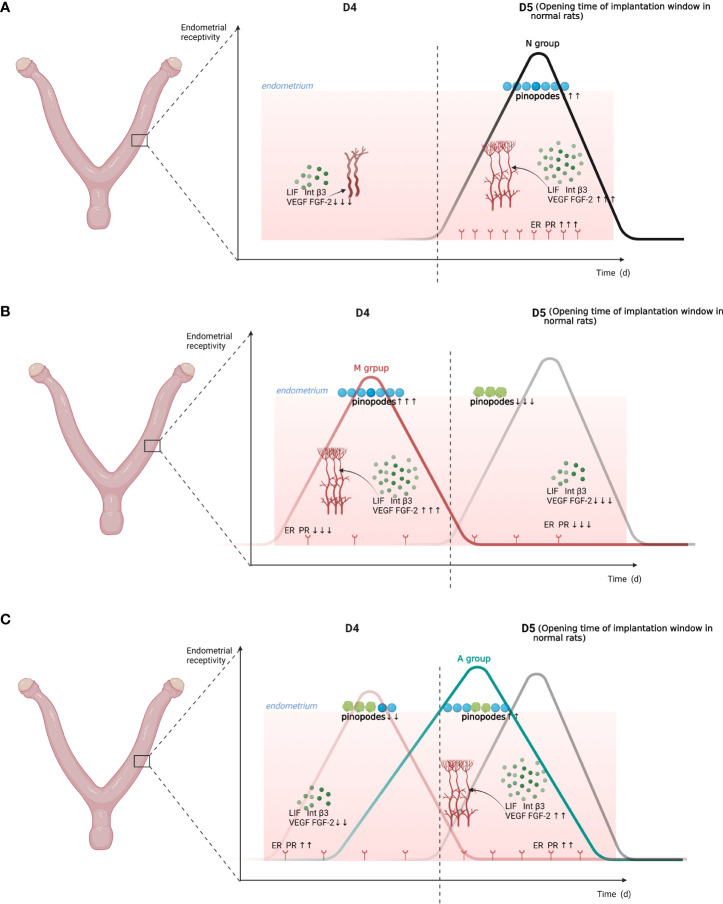

## 1 Introduction

Infertility affects up to 15% of couples worldwide (1), and more and more infertile couples are choosing *in vitro* fertilization-embryo transfer (IVF-ET) as the last resort for pregnancy. Controlled ovarian hyperstimulation (COH) is one of the most commonly used and important treatments for obtaining a large number of high-quality oocytes during IVF-ET. However, there always exit some bottlenecks in COH cycles, such as low implantation rate (only 20%–30%), high miscarriage rate, and high incidence of ovarian hyperstimulation syndrome (OHSS) (2).

Studies have shown that the steps of embryo attachment and implantation are strictly controlled by steroid hormones (3), and the super-physiological levels of estrogen, progesterone, high progesterone to estrogen ratio, unbalanced glycosylic conjugation, or human chorionic gonadotropin (HCG) induced by COH may be associated with endometrial dysplasia and unsynchronized endometrial and embryonic development, leading to implantation failure (4–6). In recent years, researchers have tried using drugs such as aspirin, heparin, and sildenafil to improve the microcirculation and trophoblast invasion during the peri-implantation period, thereby helping to enhance endometrial receptivity, but their effectiveness and safety need to be further studied (7–10).

Acupuncture has a long history in the treatment of infertility. In recent years, it has been increasingly used in ART and an increasing number of clinical studies have proved its effectiveness and safety in improving the clinical pregnancy rate (11–14). Based on the dynamic analysis of VEGF mRNA in the endometrium during the peri-implantation period in previous studies and the close relationship between endometrial angiogenesis and embryo implantation (15), we proposed a hypothesis that “COH leads to the advancement of the implantation window, the asynchrony of which with endometrial development is a key factor leading to a reduced pregnancy rate, and the mechanism of acupuncture improving the pregnancy rate of COH rats may be *via* restoring the advanced implantation window by improving the progesterone to estrogen ratio.” To verify the abovementioned hypothesis, we designed this study to further explore the curative effect of acupuncture for improving the pregnancy rate of COH rats.

## 2 Materials and methods

### 2.1 Animals and grouping

170 SPF-grade adult female virgin Wistar rats (220–250 g) and 45 healthy male Wistar rats (approximately 300 g) were purchased from the mouse Laibao Biotech Co., Ltd. and raised in the central barrier system of the Experimental Animal Center of Tongji Hospital, Tongji Medical College, Hua Zhong University of Science and Technology. Experimental rats were raised in a pathogen-free environment (20 ± 2 °C, 60 ± 5% humidity, 12 h: 12 h light/dark cycle), and were given free access to water and food (2 rats/cage). The males and females were reared in separate cages. This study was approved by the Animal Experiment Ethics Committee of Tongji Medical College, Huazhong University of Science and Technology, Wuhan, China (prove number: TJH-202009008).

After one week of adaptive feeding, the vaginal smear method was used to observe their estrus cycle every day. The 156 rats with two consecutive regular estrus cycles were randomly divided into nine groups: D4 normal group (D4N), D4 model group (D4M), and D4 acupuncture group(D4A) (n = 12 each); D5 normal group (D5N), D5 model group (D5M), and D5 acupuncture group (D5A) (n = 12 each); and D6 normal group (D6N), D6 model group (D6M), and D6 acupuncture group (D6A) (n = 28 each).

### 2.2 Reagents and main devices

Pregnant mare serum gonadotropin (PMSG) was purchased from the Hangzhou Animal Medicine Factory, China. Human chorionic gonadotrophin (HCG) was provided by Livzon Pharmaceutical Factory, Zhuhai, China. Other materials included anti-vascular endothelial growth factor (VEGF) (Santa Cruz, SC-7269), anti-fibroblast growth factor 2 (FGF-2) (Immunoway, Cat NO.YT5549), anti-estrogen receptor α (Santa Cruz, SC-787); anti-progesterone receptor A (Proteintech, Cat NO.25871-1-AP); anti-LIF, (Gentex, Cat NO.GTX11940), ITG β-3(Abclonal, Cat NO.A2542) and Evans Blue (Cat. number E8010). Main reagents and devices included quantitative real-time PCR (qRT-PCR) equipment (Applied Biosystems, USA), SYBR Green qPCR Kit (Yesen, Cat NO.11201-11203), nucleic acid protein analyzer (DU730, Beckman Coulter, USA), Mastercycler gradient PCR apparatus (Eppendorf, Germany), Nikon microimaging system (TE2000-U, Tokyo, Japan), Step-One Real-Time PCR (Applied Biosystems, California, USA), scanning electron microscope (HITACHI, SU8100, Japan), estrogen ELISA kits (No.501890, Cayman, USA), progesterone ELISA kits (Cat: ELK7894, ELK Biotechnology, China), and Odyssey infrared imaging system (licor Biosciences, USA).

### 2.3 Modeling and intervention

Our previously reported method and related literature were followed for modeling (15, 16); female rats in both model (M) and acupuncture (A) groups were intraperitoneal injected with 20 IU PMSG at 5 PM on the Day 2 of the estrus period, followed by an injection of 20 IU HCG approximately 48 h later; subsequently, the female rats were mated with male rats overnight in independent cages. At the same time, the same volume of 0.9% saline was injected into the rats in the Normal (N) group. At 8 a.m. on the day after mating, a vaginal smear was taken and a large number of sperms or vaginal plug on the vaginal smear was considered to indicate successful mating and the day was marked Day 1 (D1). Only female rats that mated successfully were included in the follow-up study. The rats in the A groups were fixed in a homemade cloth bag, and acupuncture was performed at the three acupoints of Sanyinjiao (SP6), Taichong (LR3), and Zusanli (ST36) on both sides using sterile acupuncture needles (201104, Hanyi, 0.18 × 13 mm, Beijing Hanyi Medical Instruments Co., Ltd., China) for 25 min from the day of PMSG injection to Day 4 after mating (D4). The rats in N and M groups were fixed in the same cloth bags for 25 min from the day of PMSG injection to D4 without acupuncture being performed.

### 2.4 Harvesting

At 5 PM on D4, 5, and 6, the rats were anesthetized *via* an intraperitoneal injection of 1 ml 2% sodium pentobarbital. Rats in each group were killed by an overdose of anesthesia directly after obtaining blood from the abdominal aorta in the D4 and D5 groups. After the rats were deeply anesthetized in the D6 group, the embryo implantation point was stained by a tail vein injection of 1.5 ml Evans Blue; blood was drawn and the rats were sacrificed after 10 min of dyeing; the number of implantation points was recorded in detail to calculate the pregnancy rate (number of pregnant rats/number of successfully caged rats) and number of implanted embryos (total number of implanted embryos/total number of pregnant rats). The blood samples were centrifuged (3000 rpm × 15 min) and the supernatant was collected and stored in a refrigerator at −80°C for the detection of steroid hormone levels. The uterine tissues of each group were carefully separated and preserved; a small section was put into the electron microscope fixation solution for subsequent electron microscope experiments; another small section was fixed and embedded in 4% paraformaldehyde for histochemical evaluation; the rest was freezed to −80°C in a refrigerator for subsequent experiments.

### 2.5 Scanning electron microscopy

Fresh endometrial surface tissues (approximately 1 mm^3^ in size) were quickly put into the electron microscope fixation solution, and fixed at 4°C for 2–4 h. After fixation, dehydration, infiltration, embedding, slicing, and staining, endometrial pinopodes was observed and an image was captured under a scanning electron microscope (HITACHI, SU8100, Japan).

### 2.6 Enzyme-linked immunosorbent assay

Levels of serum estrogen and progesterone were analyzed by ELISA. The sensitivity of the estrogen ELISA kits (No.501890, Cayman, USA) was approximately 20 pg/ml, and the assay has a range of approximately 0.61–10000 pg/ml. The sensitivity of progesterone ELISA kits (Cat: ELK7894, ELK Biotechnology, China) was approximately 0.55 ng/ml, and the assay has a range of 1.57–100 ng/ml. The ELISA steps were carried out in strict accordance with the instructions mentioned in the kits.

### 2.7 Immunohistochemical assay

Paraffin sections were dewaxed in xylene, placed in gradient concentrations of ethanol to recover the antigen, and then blocked with goat serum at room temperature for 20 min. Subsequently, the sections were incubated with a primary antibody (anti-estrogen receptor α, Santa Cruz Biotechnology, 1:50; anti-progesterone receptor A, Proteintech, 1:300; anti-VEGF, Santa Cruz, 1:50; anti-FGF-2, Immunoway, 1:100; anti-LIF, Gentex, 1:200; ITG β-3, Abclonal, 1:150) at 4°C overnight, rinsed with phosphate-buffered saline with Tween (PBST) five times for 5 min, and incubated with secondary antibodies (anti-estrogen receptor α, Santa Cruz Biotechnology, 1:50; anti-progesterone receptor A, Proteintech, 1:300; anti-VEGF, Proteintech, 1:50; anti-FGF-2, Bioswamp, 1:100; anti-LIF, Gentex, 1:200; ITG β-3, Absin, 1:150) at room temperature for 1 h. After washing with PBST five times for 5 min, the sections were developed using 3,3’-diaminobenzidine and stabilized using hematoxylin for approximately 3 min. Finally, the sections were dehydrated, made transparent, and sealed. The images were scanned using a nanozoomer slide scanner (Hamamastu, Japan) and observed using the NDP view2 system.

### 2.8 Western blot analysis

Endometrial tissues were homogenized and lysed in tissue protein extraction reagent, supplemented with a protease inhibitor cocktail, placed on ice for 30 min, and centrifuged at 4°C (12,000 rpm for 10 min). After the protein concentration was determined, the samples were subjected to sodium dodecyl sulfate polyacrylamide gel electrophoresis (SDS PAGE) and transferred to a PVDF membrane. The membranes were sealed with 5% skim milk at room temperature for 0.5 h, and then incubated with a primary antibody at 4 °C for 24–48 h. Antibodies include anti-estrogen receptor α (Santa Cruz Biotechnology, USA, 1:50); anti-progesterone receptor A (Proteintech, China, 1:200; anti-VEGF, Santa Cruz, China, 1:200); anti-FGF-2 (Immunoway, China, 1:500); anti-LIF (Gentex, China, 1:300); ITGβ3 (Abclonal, China, 1:500); and anti-β-actin (Proteintech, China, 1:1500). PVDF membranes were incubated with fluorescent secondary antibodies (CST, USA) on a shaking table at room temperature for 1 h. Finally, the bands were scanned using Odyssey infrared imaging system (licor Biosciences, USA).

### 2.9 Real-time PCR

Total RNA was extracted from the endometrial tissue using Trizol reagent (Takara, Japan), according to the manufacturer’s instructions. After the RNA concentration was determined, the cDNA was synthesized with a reverse transcription reagent (Yesen, China). Quantitative real-time PCR (qRT-PCR) (Applied Biosystems, USA) was performed using SYBR Green qPCR Kit (Yesen, China). The 2^−ΔΔCT^ method was used for data analysis. The sequence is in the **Table 1**.

**Table 1:** Primer information of the LIF, ITGβ3, VEGF, FGF-2, ERα and PRα mRNA sequences.

Name	Primer Information	Base sequence	annealing temperature	GC%	Length
LIF	R-lif-S	GGGATTGTGCCCCTACTGCTC	62.8	61.9	152
	R-lif-A	CCGTTGAGTTGAGCCAGTTGAC	61.94	54.55	
ITGB3	R-itgB3(2)-S	ACCGTTTCTGCCGAGATGAC	60.39	55	322
	R-itgB3(2)-A	CATTTGGCTCTGGCTCGTTC	59.55	55	
VEGF	R-VEGF(1)-S	GCACTGGACCCTGGCTTTACT	62.34	57.14	102
	R-VEGF(1)-A	AACTTCACCACTTCATGGGCTTT	60.95	43.48	
FGF	R-FGF2-S	GAGAAGAGCGACCCACACGT	59.7	60	232
	R-FGF2-A	CAGTTCGTTTCAGTGCCACATAC	59.9	47.8	
ERα	R-ERα(1)-S	GTTTGCTCCTAACTTGCTCTTGG	60.06	47.83	191
	R-ERα(1)-A	TCAAGGTGCTGGATAGAAATGTG	58.74	43.48	
PRα	R-PRα-S	TAGTCAAATGGTCTAAGTCTCTGCC	60.11	44	215
	R-PRα-A	GGTAAGGCACAGCGAGTAGAATG	61.29	52.17	
GAPDH	R-GAPDH-S	CTGGAGAAACCTGCCAAGTATG	58.99	50	138
	R-GAPDH-A	GGTGGAAGAATGGGAGTTGCT	60.27	52.38	

LIF, Leukemia inhibitory factor; ITGβ3, Integrin β3; VEGF, Vascular endothelial growth factor; FGF-2, Fibroblast growth factor 2; ERα, Estrogen receptorα; PRα, Progesterone receptorα.

### 2.10 Statistical analysis

IBM SPSS 20.0 was used for statistical analysis. Continuous data for normal distribution are expressed as means ± SD and categorical data are expressed as percentages (%). Differences were compared using one-way analysis of variance. If the variance was uniform, the LSD test was used, whereas if it was uneven, Dunnett’s T3 test was used. P value < 0.05 was considered to be statistically significant.

## 3 Results

### 3.1 Comparison between the mating rate and pregnancy rate of rats in each group

**Figure 1:**
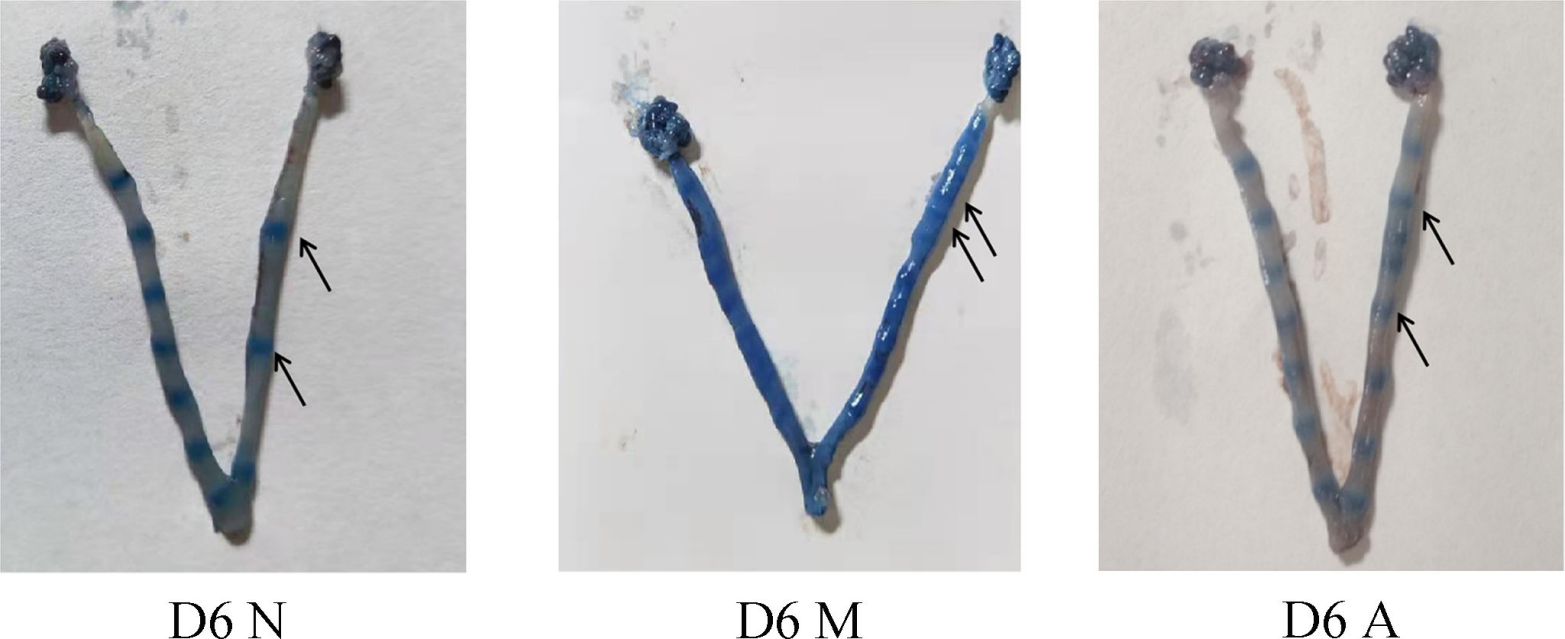
Uterus of embryo implantation in each group on D6. D, The embryo day; D6N, D6 normal group; D6M, D6 model group; D6A, D6 acupuncture group.

A large number of spermatozoa or vaginal suppositories on vaginal smears were regarded to indicate successful mating. In addition, only rats in the D6 group were included for calculating the pregnancy rate. In all, 42 rats were injected with Evans blue through the tail vein on Day 6 to determine whether they were pregnant (**Table 2**). Compared with the N group, the M group showed a significantly lower pregnancy rate (*P* < 0.01). Further, compared with the M group, the A group showed a significantly higher pregnancy rate (*P* < 0.05). But the specific number of pregnant embryos in each rat could not be accurately counted. Accordingly, the number of embryos was not calculated. According to Evans blue staining, the M group had more embryos than the N group (**Figure 1**).

**Table 2:** Mating rate of rats in each group and pregnancy rate of D6 rats.

Group	Mating Rate (%)	Pregnancy Rate on D6 (%)
N	100 (52/52)	100 (14/14)
M	90.38 (47/52)	42.86 (6/14)^**^
A	94.23 (49/52)	64.29 (9/14)^#^

The value is expressed as mean ± SD. *or** represents that there is significant difference between M and N groups (P<0.05 or P<0.01), #or ## represents that there is significant difference between A and M groups (P<0.05 or P<0.01), D6: The embryo day 6; N: Normal group; M: Model group; A: Acupuncture group.

### 3.2 Comparison of pinopodes in rat endometrium

**Figure 2:**
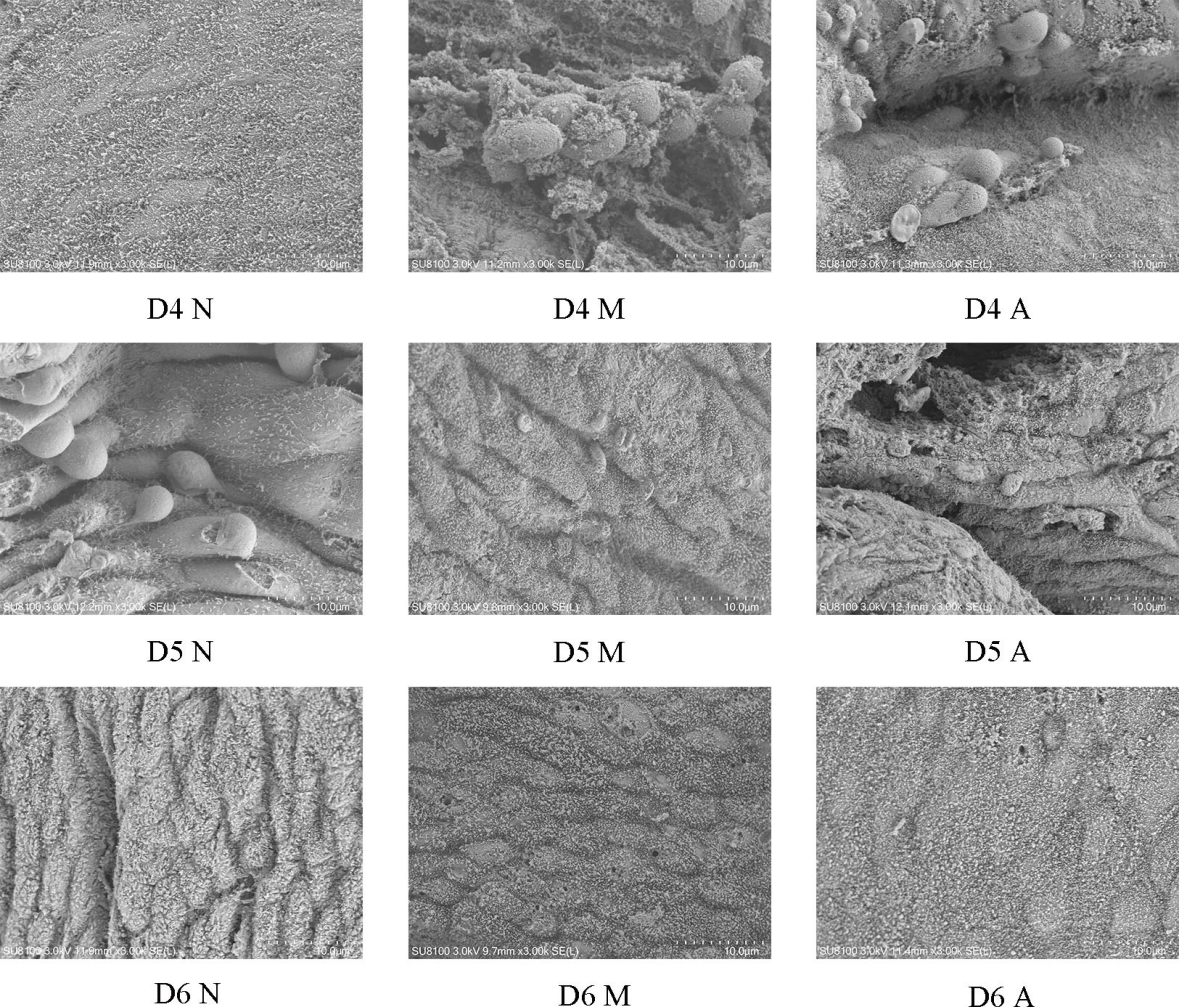
The expression of pinocytosis in endometrium of each group under scanning electron microscope (3000*). D, The embryo day; D4 N, D4 normal group; D4 M, D4 model group; D4 A, D4 acupuncture group; D5 N, D5 normal group; D5 M, D5 model group; D5 A, D5 acupuncture group; D6 N, D6 normal group; D6 M, D6 model group; D6 A, D6 acupuncture group.

The ultrastructural results of endometrium on D4, D5, and D6 were observed under a scanning electron microscope (3000×). There was no pinopode or swollen microvilli on the surface of endometrium in the D4N group, a large number of pinopodes in the D4M group, and obvious pinopodes in the D4A group, but the number of pinopodes in the D4A group was less than that in the D4M group. There were a large number of mature pinopodes on the endometrial surface the rats in the D5N group, a small number of atrophic pinopodes in the D5M group, and a small number of mature or atrophic satiety pinopodes in the D5A group, and the number of pinopodes observed in the D5M group was less than that observed in the in D5A group. There were no pinopodes in the D6N, M, and A groups (**Figure 2**).

### 3.3 Comparison of protein and gene expressions of LIF, integrin β3, VEGF, and FGF-2 in the endometrium

**Figure 3:**
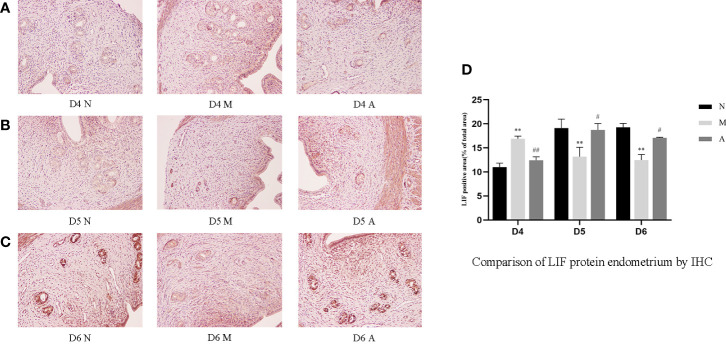
The expression of endometrial LIF protein: **(A)** D4; **(B)** D5; **(C)** D6; and **(D)** comparison of LIF protein by mean gray value (n=3). *or** represents that there is significant difference between M and N groups (*P* < 0.05 or *P* < 0.01), #or ## represents that there is significant difference between A and M groups (*P* < 0.05 or *P* < 0.01). Original magnification *200. LIF, Leukemia inhibitory factor; IHC, Immunohistochemistry; D, The embryo day; D4 N, D4 normal group; D4 M, D4 model group; D4 A, D4 acupuncture group; D5 N, D5 normal group; D5 M, D5 model group; D5 A, D5 acupuncture group; D6 N, D6 normal group; D6 M, D6 model group; D6 A, D6 acupuncture group; N, Normal group; M, Model group; A, Acupuncture group.

**Figure 4:**
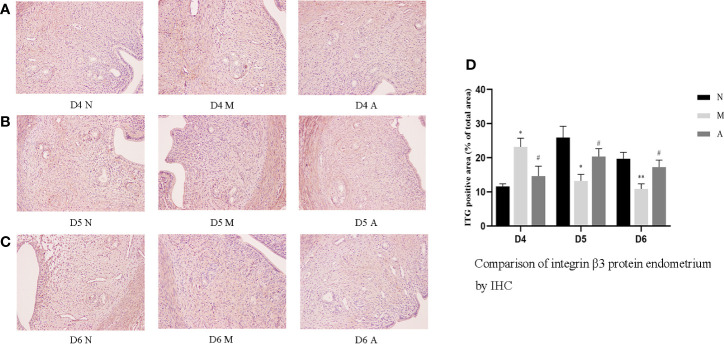
The expression of endometrial integrin β3 protein: **(A)** D4; **(B)** D5; **(C)** D6; and **(D)** comparison of integrin β3 protein by mean gray value (n=3). *or** represents that there is significant difference between M and N groups (*P* < 0.05 or *P* < 0.01), #or ## represents that there is significant difference between A and M groups (*P* < 0.05 or *P* < 0.01). Original magnification *200. IHC, Immunohistochemistry; D, The embryo day; D4 N, D4 normal group; D4 M, D4 model group; D4 A, D4 acupuncture group; D5 N, D5 normal group; D5 M, D5 model group; D5 A, D5 acupuncture group; D6 N, D6 normal group; D6 M, D6 model group; D6 A, D6 acupuncture group; N, Normal group; M, Model group; A, Acupuncture group.

**Figure 5:**
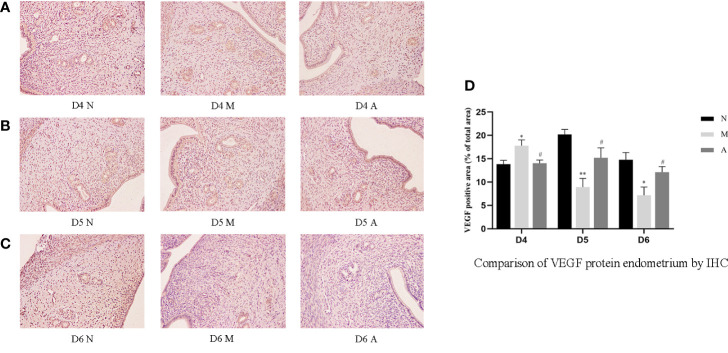
The expression of endometrial VEGF protein: **(A)** D4; **(B)** D5; **(C)** D6; and **(D)** comparison of VEGF protein by mean gray value (n=3). *or** represents that there is significant difference between M and N groups (*P* < 0.05 or *P* < 0.01), #or ## represents that there is significant difference between A and M groups (*P* < 0.05 or *P* < 0.01). Original magnification *200. VEGF, Vascular endothelial growth factor; IHC, Immunohistochemistry; D, The embryo day; D4 N, D4 normal group; D4 M, D4 model group; D4 A, D4 acupuncture group; D5 N, D5 normal group; D5 M, D5 model group; D5 A, D5 acupuncture group; D6 N, D6 normal group; D6 M, D6 model group; D6 A, D6 acupuncture group; N, Normal group; M, Model group; A, Acupuncture group.

**Figure 6:**
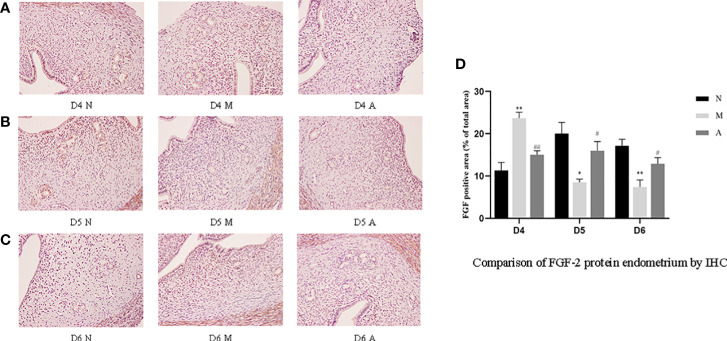
The expression of endometrial FGF-2 protein: **(A)** D4; **(B)** D5; **(C)** D6; and **(D)** comparison of VEGF protein by mean gray value (n=3). *or** represents that there is significant difference between M and N groups (*P* < 0.05 or *P* < 0.01), #or ## represents that there is significant difference between A and M groups (*P* < 0.05 or *P* < 0.01). Original magnification *200. FGF-2, Fibroblast growth factor 2; IHC, Immunohistochemistry; D, The embryo day; D4 N, D4 normal group; D4 M, D4 model group; D4 A, D4 acupuncture group; D5 N, D5 normal group; D5 M, D5 model group; D5 A, D5 acupuncture group; D6 N, D6 normal group; D6 M, D6 model group; D6 A, D6 acupuncture group; N, Normal group; M, Model group; A, Acupuncture group.

**Figure 7:**
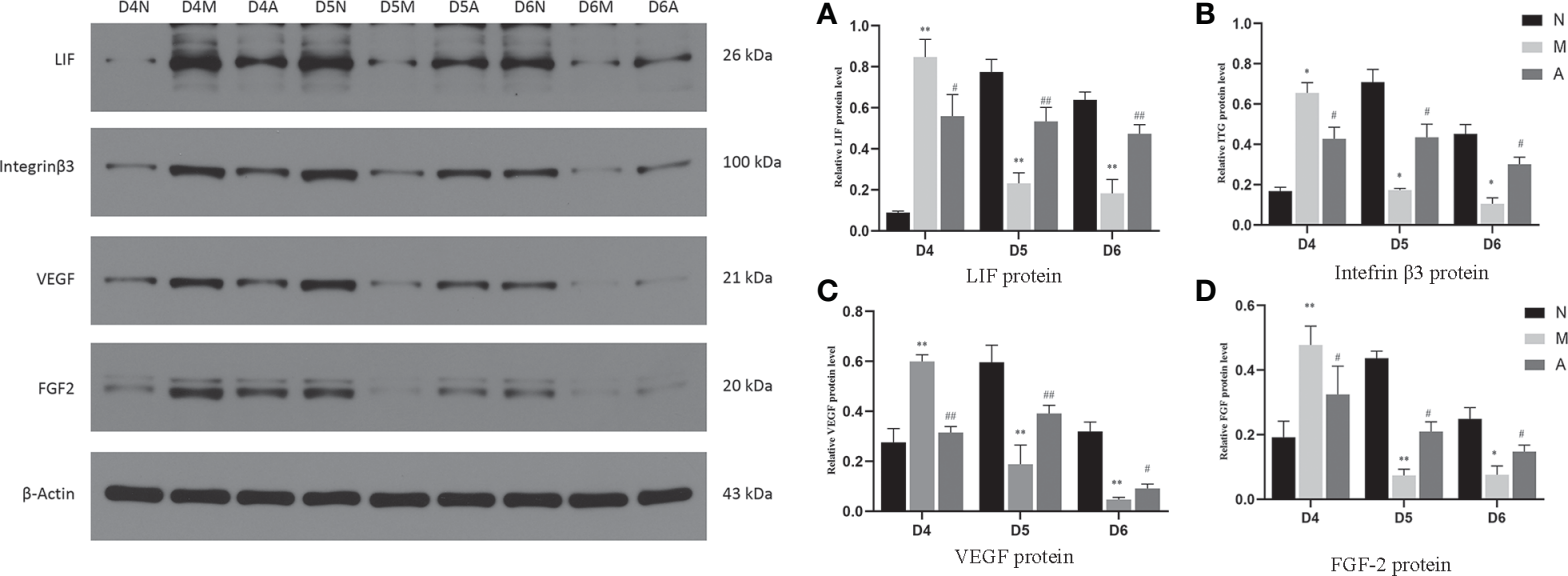
The expression of LIF **(A)**, integrin β3 protein **(B)**, VEGF **(C)**, and FGF-2 **(D)** protein in the endometrium by West-bolt. *or** represents that there is significant difference between M and N groups (*P* < 0.05 or *P* < 0.01), #or ## represents that there is significant difference between A and M groups (*P* < 0.05 or *P* < 0.01). LIF, Leukemia inhibitory factor; VEGF, Vascular endothelial growth factor; FGF-2, Fibroblast growth factor 2; D, The embryo day; N, Normal group; M, Model group; A, Acupuncture group.

Immunohistochemistry (IHC) results showed that LIF, integrin β3, VEGF, and FGF-2 proteins were mostly expressed in the luminal and glandular epithelia, stroma, and myometrium of the endometrium. The expression of endometrial LIF, integrin β3, VEGF, and FGF-2 protein by IHC (**Figures 3**–**6**) were consistent with the WB (**Figure 7**).

On D4, the expressions of LIF, integrin β3, VEGF, and FGF-2 protein in the endometrium of rats in the D4M group were significantly increased compared with those in the endometrium of rats in the D4N group (*P* < 0.05, *P* < 0.01), whereas the expressions in the D4A group were significantly decreased compared with those in the D4M group (*P* < 0.05, *P* < 0.01). On D5 and D6, the expressions of LIF, integrin β3, VEGF, and FGF-2 protein in the endometrium of rats in the M group were significantly decreased compared with those in the endometrium of rats in the N group (*P* < 0.05, *P* < 0.01), whereas the expressions in the A group were significantly increased compared with those in the M group(*P* < 0.05, *P* < 0.01) (**Figure 7**).

**Figure 8:**
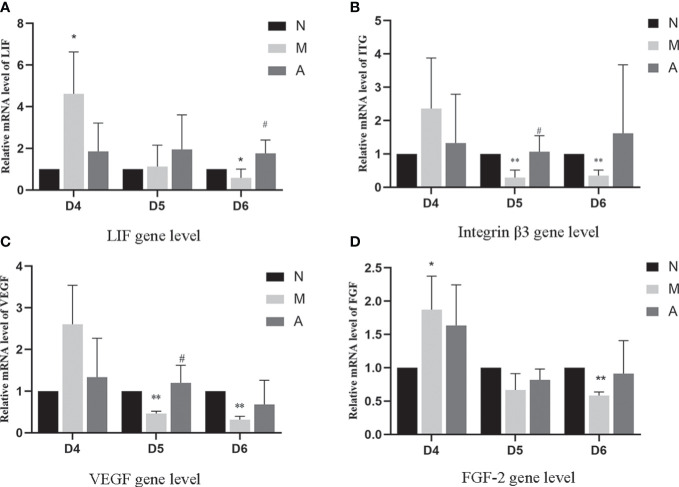
The expression of LIF **(A)**, integrin β3 protein **(B)**, VEGF **(C)**, and FGF-2 **(D)** mRNA levels in the endometrium (n=5). *or** represents that there is significant difference between M and N groups (*P* < 0.05 or *P* < 0.01), #or ## represents that there is significant difference between A and M groups (*P* < 0.05 or *P* < 0.01). LIF, Leukemia inhibitory factor; VEGF, Vascular endothelial growth factor; FGF-2, Fibroblast growth factor 2; mRNA, Messenger RNA; D, The embryo day; N, Normal group; M, Model group; A, Acupuncture group.

PCR results showed that the mRNA expression of LIF and FGF in the D4M group was significantly higher than that in the D4N group (*P* < 0.05), and acupuncture could alleviate this change, but there was no significant difference between D4A and D4M groups (*P* > 0.05). In addition, there was no difference in the mRNA expression of integrin β3 and VEGF among D4N, D4M, and D4A groups (*P* > 0.05). On D5, the mRNA expression of integrin β3 and VEGF in the D5M group was significantly lower than that in the D5N group (*P* < 0.01), but acupuncture could significantly reverse this change (*P* < 0.05). At the same time, there was no significant difference of the mRNA expression of LIF and FGF among D5N, D5M, and D5A groups (*P* > 0.05). On D6, the mRNA expression of LIF, integrin β3, VEGF and FGF-2 in the D6M group was significantly decreased compared with that in the D6N group (*P* < 0.05 or *P* < 0.01), while acupuncture could increase the mRNA expression of LIF compared with that in the D6M group (*P* < 0.05). As for the mRNA expression of integrin β3, VEGF and FGF-2, although acupuncture could increase their expression, but no significant difference was observed between the D6A and D6M groups (*P* > 0.05) (**Figure 8**).

### 3.4 Comparison of serum estrogen and progesterone levels and endometrial estrogen and progesterone receptor protein and gene levels

**Figure 9:**
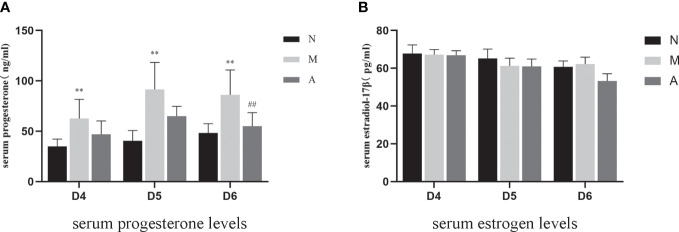
The expression of serum progesterone **(A)** and estrogen **(B)** levels (n=7). *or** represents that there is significant difference between M and N groups (*P* < 0.05 or *P* < 0.01), #or ## represents that there is significant difference between A and M groups (*P* < 0.05 or *P* < 0.01). D, The embryo day; N, Normal group; M, Model group; A, Acupuncture group.

There was no significant difference in D4, D5 and D6 estrogen levels among N, M, and A groups. Compared with the N group, the M group showed significantly higher levels of progesterone on D4, D5, and D6 (*P* < 0.05, *P* < 0.01). There were no significant differences in progesterone levels between the M and A groups on D4 and D5 (*P* > 0.05), whereas the levels were significantly lower on D6 in the A group compared with M group (*P* < 0.05) (**Table 3**, **Figure 9**).

**Table 3:** The expression of serum progesterone and estrogen levels.

	Group	D4	D5	D6
Progesterone	N	34.99 ± 2.52 (n=8)	40.60± 3.55 (n=8)	48.35 ±3.19 (n=8)
	M	62.71± 6.72 (n=8)^*^	91.54 ± 9.48 (n=8) ^**^	86.17± 8.70(n=8) ^**^
	A	46.89 ±13.23 (n=8)	64.94± 3.43 (n=8)	55.05 ± 4.75 (n=8) ^#^
Estrogen	N	67.84 ± 1.59 (n=8)	65.22 ± 1.75 (n=8)	60.80 ± 1.06(n=8)
	M	67.22 ± 2.66 (n=8)	61.22± 1.46 (n=8)	62.26 ± 1.28 (n=8)
	A	66.92 ± 2.37 (n=8)	60.94± 1.40 (n=8)	56.81 ± 2.21 (n=8)

The value is expressed as mean ± SD;*or** represents that there is significant difference between M and N groups (P < 0.05 or P < 0.01), #or ## represents that there is significant difference between A and M groups (P < 0.05 or P < 0.01), D4, The embryo day 4; D5, The embryo day 5; D6, The embryo day 6; N, Normal group; M, Model group; A, Acupuncture group.

**Figure 10:**
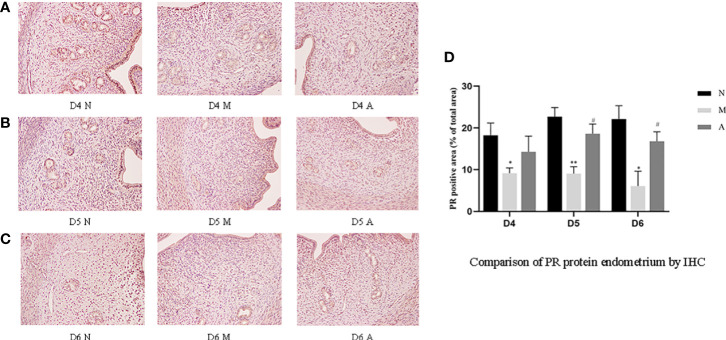
The expression of endometrial PR protein: **(A)** D4; **(B)** D5; **(C)** D6; and **(D)** comparison of PR protein by mean gray value (n=3). *or** represents that there is significant difference between M and N groups (*P* < 0.05 or *P* < 0.01), #or ## represents that there is significant difference between A and M groups (*P* < 0.05 or *P* < 0.01). Original magnification *200. PR: Progesterone receptor; IHC, Immunohistochemistry; D, The embryo day; D4 N, D4 normal group; D4 M, D4 model group; D4 A, D4 acupuncture group; D5 N, D5 normal group; D5 M, D5 model group; D5 A, D5 acupuncture group; D6 N, D6 normal group; D6 M, D6 model group; D6 A, D6 acupuncture group; N, Normal group; M, Model group; A, Acupuncture group.

**Figure 11:**
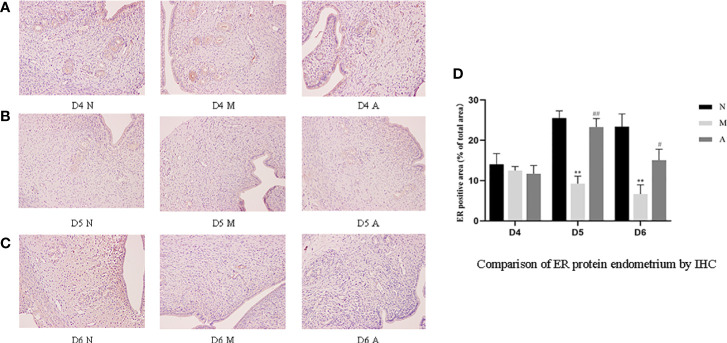
The expression of endometrial ER protein: **(A)** D4; **(B)** D5; **(C)** D6; and **(D)** comparison of ER protein by mean gray value (n=3). *or** represents that there is significant difference between M and N groups (*P* < 0.05 or *P* < 0.01), #or ## represents that there is significant difference between A and M groups (*P* < 0.05 or *P* < 0.01). Original magnification *200. ER, Estrogen receptor; IHC, Immunohistochemistry; D, The embryo day; D4 N, D4 normal group; D4 M, D4 model group; D4 A, D4 acupuncture group; D5 N, D5 normal group; D5 M, D5 model group; D5 A, D5 acupuncture group; D6 N, D6 normal group; D6 M, D6 model group; D6 A, D6 acupuncture group; N, Normal group; M, Model group; A, Acupuncture group.

**Figure 12:**
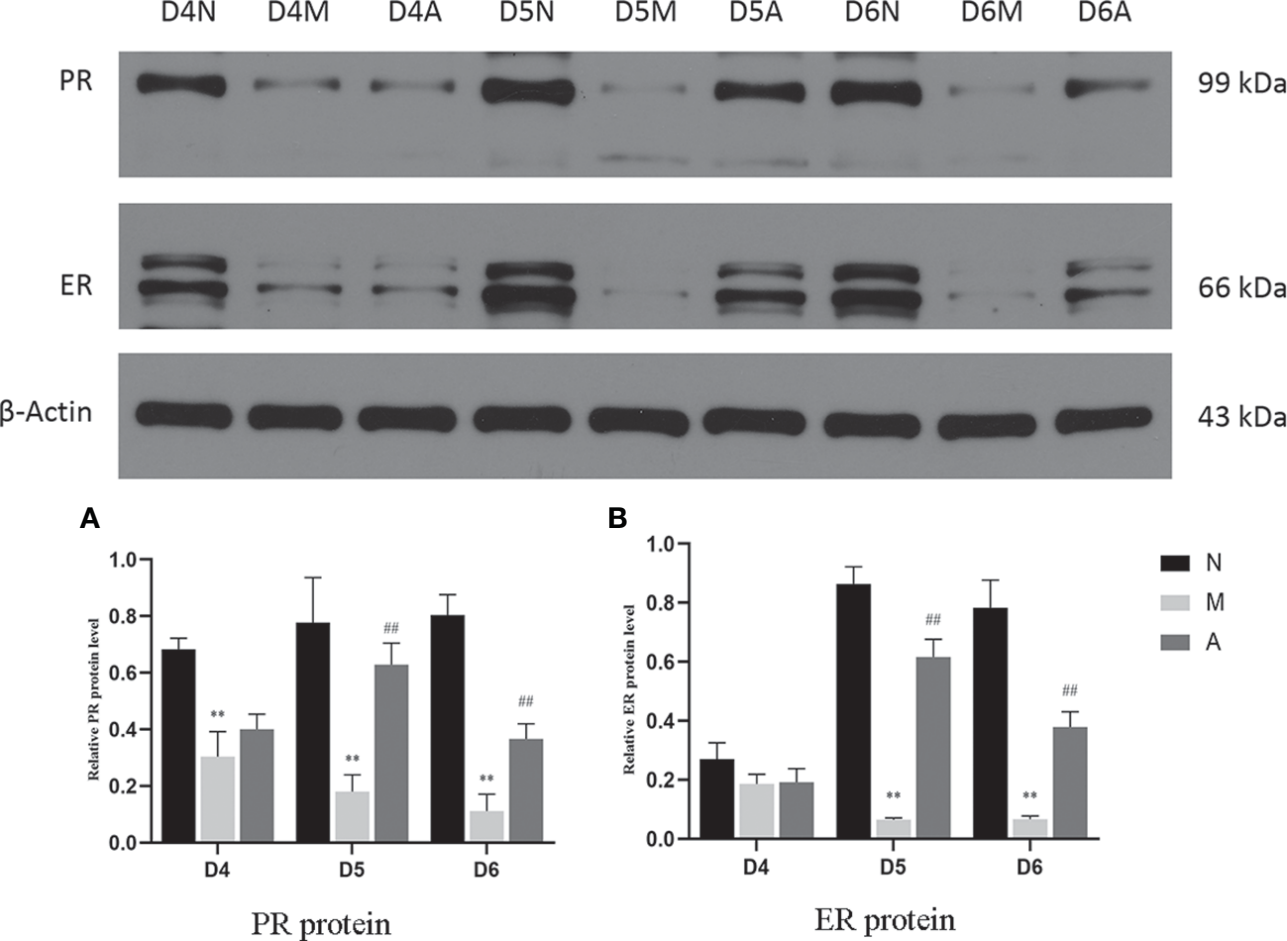
The expression of PR **(A)** and ER **(B)** protein in the endometrium by West-bolt. *or** represents that there is significant difference between M and N groups (*P* < 0.05 or *P* < 0.01), #or ## represents that there is significant difference between A and M groups (*P* < 0.05 or *P* < 0.01). PR, Progesterone receptor; ER, Estrogen receptor; D, The embryo day; N, Normal group; M, Model group; A, Acupuncture group.

IHC results showed that progesterone receptor (PR) and estrogen receptor α (ERα) mainly expressed in glandular epithelium and luminal epithelium of the endometrium, and a small amount was expressed in stromal cells. The epression of endometrial PR and Erα proteins by IHC (**Figures 10**, **11**) were consistent with the WB (**Figure 12**).

PR protein levels in the D4M, D5M, and D6M groups were significantly lower than those in the corresponding N groups (*P* < 0.01). The PR protein levels in D5A and D6A groups were significantly higher than those in the corresponding M groups (*P* < 0.01). With regard to ERα, there was no significant difference between the M and N Group on D4 (*P* > 0.05). Compared with the D5N group, the D5M group showed significantly lower ERα protein levels (*P* < 0.01), whereas the D5A group showed significantly higher levels compared with the D5M group (*P* < 0.01). The expression trend for D6 ERα protein level was consistent with that observed for D5 (**Figure 12**).

PCR results showed that the mRNA level of ERα on the endometrium in the D4M group was increased compared with that in the D4N group (*P* < 0.05), whereas no significant difference was observed between D4A and D4M (*P* > 0.05). The expression trend for ERα protein levels in the D5M group was decreased compared with that in the D5N group (*P* < 0.05). There was no significant difference among the three D6 groups. The mRNA level of PR on the endometrium in D6M groups was decreased compared with that in the D6N group (*P* < 0.01); however, no significant difference was observed among other groups (*P* > 0.05) (**Supplementary Image 1**).

## 4 Discussion

The results of pregnancy in this experiment are consistent with our previous findings (15). The overall pregnancy rate of COH rats is significantly reduced but the number of implanted embryos in pregnant rats is higher than normal rats, and acupuncture can increase the pregnancy rate and decrease the number of implanted embryos. Consistent with many previous studies, we speculated that the main reason for the decline in pregnancy rate after COH may be the decrease in the endometrial receptivity (17, 18). Our research results also confirm this viewpoint. However, interestingly, based on our research results, we believe that the damage caused by COH may be not only the ability of endometrium to withstand embryo implantation, which is generally considered and concerned by most researchers, but also the advance of embryo implantation window.

The endometrial receptivity and the quality of embryos are the key factors responsible for a successful pregnancy. The recent view is that endometrial receptivity is a complex process that provides the embryo with the opportunity to attach, invade, and develop, culminating in a new individual and continuation of the species (19). There are many factors affecting endometrial receptivity, and the opening of the implantation window is generally considered to be an important part of the complex process of endometrial receptivity (19). The implantation window refers to a period of close interaction between high-quality blastocysts required for embryo implantation and the endometrium that can accept embryo implantation and this time is usually very short (20). At present, the commonly used indicators for evaluating endometrial receptivity include ultrasound indicators (such as endometrial thickness, type, and subendometrial blood flow), cellular level indicators, and molecular level indicators (such as levels of integrated hormones, LIF, estrogen and progesterone) (15, 19–22). This study is to verify our hypothesis and the therapeutic mechanism of acupuncture through the dynamic detection of these cellular level indicators and molecular level indicators during the peri-implantation period.

The opening of the implantation window in normal rats occurs generally on about D5 after successful mating (23). Although pinopodes are rather disputed during the recent years, its number and shape are still closely associated with the endometrial receptivity and implantation window (24–26). In our experiment, it was observed that the normal rats showed no pinopodes in the endometrium on D4, there were a large number of mature pinopodes on D5, and the pinopodes was atrophied on D6. However, COH rats had a large number of mature pinopodes on D4, but they began to atrophy and subside on D5. In contrast, acupuncture could significantly pull back the forward movement of pinopodes, indicating that COH may lead to the forward movement of the implantation window, and acupuncture could restore the early implantation window.

Integrin β3 is an important molecule in the process of an embryo’s initial attachment and cell adhesion, which can guide the adhesion of trophoblasts and subsequent implantation (21). In addition, integrin β3 is closely associated with the time order of endometrial receptivity and synchronized with the opening of the implantation window (27). Outcomes in IVF were poor in women that were in phase histologically but lacked the integrin β3 (19, 28). LIF is the first and most durable endometrial protein recognized as essential for implantation (29, 30). As a cytokine of the IL-6 family, LIF utilizes a receptor that consists of the LIF receptor β and gp130, and initiates the signal transduction cascades that phosphorylates STAT3 through Janus kinases (JAK) and signal transducer and activation of transcription protein (STAT) pathway in the uterus, and plays a very important role in implantation (21, 30–32). LIF is expressed on the endometrium throughout the menstrual cycle and increases significantly from mid-secretory to late-secretory which is a finite period defined as the implantation window (30, 33, 34). It has been reported that blastocysts in LIF-knockout mice cannot implant successfully (35). Studies have proved that acupuncture can increase Integrin β3 and LIF expression to improves pregnancy outcomes in rats with thin endometrium (25), in PCOS rats (26), and in rats of implantation failure (36). We further continuously and dynamically observed these indicators at three time points: before, during, and after implantation (D4, D5, and D6) to explore the dynamic changes of implantation window and the mechanism of acupuncture. Our results show that the levels of LIF and integrin β3 were first increase and then decrease in the normal group, reaching its peak level on D5. But in the model group, they were significantly higher than those in the normal group on D4, but decreased sharply on D5 and D6 days, reaching its peak level on D4. Acupuncture could restore the trend of early expression caused by COH to a certain extent. Our results is consistent with Fang’s which showed that electroacupuncture improves endometrial receptivity through increasing LIF expression in COH rats (37). Although the results of LIF and integrin β3 at the gene level were not completely consistent those at the protein level, we got the same results at the protein level with regard to the expression of LIF mRNA on D4 and integrin β3 mRNA on D5 and D6. However, there was no statistical difference in the other comparison. The unknown cascade effect in gene-protein expression may be the reason.

During embryo implantation, angiogenesis is a key factor that determines the endometrial receptivity and the opening of the implantation window. Therefore, angiogenesis markers such as VEGF and FGF are also considered to be closely related to the opening of the uterine implantation window and are used as one of the potential molecular markers (38). Xing et al. showed that acupuncture increased VEGF gene/protein expression in the endometrium of PCOS rat to improve endometrial receptivity (26). Our results show that the continuous dynamic expression of VEGF and FGF-2 protein were basically consistent with integrin β3 and LIF. The gene and protein level trends of FGF-2 and VEGF were consistent on D4 and D6, which is also consistent with our previous experiments (15). However, on D5, only VEGF mRNA expression was consistent with the protein expression, and there was no statistical difference in FGF-2 mRNA expression in each group. The reason for this may also be related to the unknown cascade effect in the process of gene-to-protein expression.

In a nutshell, the above mentioned continuous dynamic experimental observation results from the cellular level to the molecular level systematically confirm the first half of our hypothesis. The decrease in LIF, integrin β3, FGF, and VEGF indices of COH rats on D5 and D6 may be an artifact caused by withdrawal after the peak on D4. In addition, although the pregnancy rate of COH rats decreased on D6, there were still more implantation sites than the normal group, which also may suggest that the ability of the endometrium to accommodate embryo implantation after COH may not be much impaired. The advanced implantation window caused by COH may be an important factor associate with the reduction in pregnancy rate in COH rats, and the effective role of acupuncture may be to restore the implantation window to normal and improve the local angiogenesis of the endometrium during the peri-implantation period, thereby improving the endometrial receptivity and finally improving pregnancy outcomes. In addition, to explain the further mechanism of acupuncture, we tested serum estrogen and progesterone levels and endometrium ER and PR.

Estrogen and progesterone are upstream targets of many links in the implantation mechanism, which can activate multiple downstream links to guide the structural and functional remodeling in the process of implantation (17). The peak of estrogen can initiate embryo implantation, and progesterone could down-regulate ER and PR receptors in the endometrium (39) and maintain stromal decidualization of the endometrium (40). Down-regulation of ER were proved to be implicated in abnormal expression of other endometrial biomarkers, such as Integrin β3 (19). Hence, the endometrial receptivity is closely associated with the level of peripheral blood steroid hormones levels and ER and PR (41). Therefore, some scholars have proposed that the body produces too much progesterone after COH, resulting in the imbalance of the progesterone to estrogen ratio, which further leads to the unsynchronized opening of the implantation window and endometrial development, leading to implantation failure (4, 5). Our results showed that although there was no significant difference in the serum estrogen levels in rats in each group, the progesterone level in the model group rats increased significantly during the three-day peri-implantation period, and acupuncture reduced the progesterone levels. The changing trend of estrogen and progesterone levels in our COH rats is consistent with the results in the COH mouse model reported by Song et al. (42). On D5 and D6, the expression of ERα and PR protein and gene levels in the model group significantly decreased; however, acupuncture could increase the expressions of ERα and PR. We speculate that the super-physiological dose of progesterone disrupts the balance of estrogen and progesterone, and at the same time, affects the expressions of ERα and PR, which in turn leads to the advancement of the embryo implantation window and unsynchronized development of the endometrium. Acupuncture may play a certain role in improving all of the above links.

However, our research also has certain limitations. At present, acupuncture is generally regarded as a mechanism that exerts benefits through multi-target and multi-step overall regulation. Our research shows that acupuncture could reduce the progesterone to estrogen ratio, and make the implantation window return to normal. However, our results may still be a phenomenon in which acupuncture takes effect. The specific efficacy mechanisms of acupuncture may involve many complexities including angiogenesis, immune tolerance regulation, and endocrine regulation, among others. The specific mechanisms of acupuncture for improving the pregnancy rate in IVF-ET need to be confirmed by future research.

## 5 Conclusions

In this study, we continuously and dynamically observed markers related to endometrial receptivity at three time points: before, during, and after implantation (D4, D5, and D6), and found that the embryo implantation window moves forward after COH and asynchrony with the development of the endometrium may be important factors associated with a reduced pregnancy rate in COH rats. The effective targets of acupuncture could not only improve the local angiogenesis of the endometrium, progesterone to estrogen ratio, and the levels of ERα and PR, improving the ability of the endometrium to withstand embryo implantation, but also make the implantation window tend to return to normal.

## Data availability statement

The raw data supporting the conclusions of this article will be made available by the authors, without undue reservation.

## Ethics statement

The animal study was reviewed and approved by Animal Experiment Ethics Committee of Tongji Medical College, Huazhong University of Science and Technology, Wuhan, China (prove number: TJH-202009008).

## Author contributions

HD contributed to the conception of this article. RH wrote the manuscript. RH and YH completed the study, RH, HD, MZ, GH and YS revised the manuscript. RH, KS and XW designed and illustrated the figures. RH, HD, MZ, GH and YS performed the literature search and interpretation. RH, YH, YS, XW, KS, GH, MZ and HD reviewed the manuscript. All authors contributed to the article and approved the submitted version.

## References

[1] Infertility workup for the women’s health specialist: Acog committee opinion, number 781. Obstetrics gynecol (2019) 133:e377–e84. doi: 10.1097/aog.0000000000003271 31135764

[2] Hill MJ Levens ED Levy G Ryan ME Csokmay JM DeCherney AH. The use of recombinant luteinizing hormone in patients undergoing assisted reproductive techniques with advanced reproductive age: A systematic review and meta-analysis. Fertility sterility (2012) 97:1108–14.e1. doi: 10.1016/j.fertnstert.2012.01.130 22365075

[3] Cha J Sun X Dey SK. Mechanisms of implantation: Strategies for successful pregnancy. Nat Med (2012) 18:1754–67. doi: 10.1038/nm.3012 PMC6322836 23223073

[4] Tavaniotou A Smitz J Bourgain C Devroey P. Ovulation induction disrupts luteal phase function. Ann New York Acad Sci (2001) 943:55–63. doi: 10.1111/j.1749-6632.2001.tb03790.x 11594558

[5] Bentin-Ley U. Relevance of endometrial pinopodes for human blastocyst implantation. Hum Reprod (Oxford England) (2000) 15 Suppl 6:67–73. 11261485

[6] Kliman HJ Frankfurter D. Clinical approach to recurrent implantation failure: Evidence-based evaluation of the endometrium. Fertility sterility (2019) 111:618–28. doi: 10.1016/j.fertnstert.2019.02.011 30929719

[7] Yu X Gao C Dai C Yang F Deng X. Endometrial injury increases expression of hypoxia-inducible factor and angiogenesis in the endometrium of women with recurrent implantation failure. Reprod biomed Online (2019) 38:761–7. doi: 10.1016/j.rbmo.2018.12.027 30885666

[8] Groom KM David AL. The role of aspirin, heparin, and other interventions in the prevention and treatment of fetal growth restriction. Am J obstetrics gynecol (2018) 218:S829–s40. doi: 10.1016/j.ajog.2017.11.565 29229321

[9] Mekinian A Cohen J Alijotas-Reig J Carbillon L Nicaise-Roland P Kayem G. Unexplained recurrent miscarriage and recurrent implantation failure: Is there a place for immunomodulation? Am J Reprod Immunol (New York NY: 1989) (2016) 76:8–28. doi: 10.1111/aji.12493 26847715

[10] Fan J Zhong Y Chen C. Combined treatment of prednisone and aspirin, starting before ovulation induction, may improve reproductive outcomes in ana-positive patients. Am J Reprod Immunol (New York NY: 1989) (2016) 76:391–5. doi: 10.1111/aji.12559 27618792

[11] Paulus WE Zhang M Strehler E El-Danasouri I Sterzik K. Influence of acupuncture on the pregnancy rate in patients who undergo assisted reproduction therapy. Fertility sterility (2002) 77:721–4. doi: 10.1016/s0015-0282(01)03273-3 11937123

[12] Xie ZY Peng ZH Yao B Chen L Mu YY Cheng J. The effects of acupuncture on pregnancy outcomes of *in vitro* fertilization: A systematic review and meta-analysis. BMC complementary Altern Med (2019) 19:131. doi: 10.1186/s12906-019-2523-7 PMC6570865 31200701

[13] Ng EH So WS Gao J Wong YY Ho PC. The role of acupuncture in the management of subfertility. Fertility sterility (2008) 90:1–13. doi: 10.1016/j.fertnstert.2008.02.094 18440533

[14] Manheimer E Zhang G Udoff L Haramati A Langenberg P Berman BM. Effects of acupuncture on rates of pregnancy and live birth among women undergoing *in vitro* fertilisation: Systematic review and meta-analysis. BMJ (Clinical Res ed) (2008) 336:545–9. doi: 10.1136/bmj.39471.430451.BE PMC2265327 18258932

[15] Dong H Zhong Z Chen W Wu X Zhang Q Huang G. Effect of acupuncture on endometrial angiogenesis and uterus dendritic cells in coh rats during peri-implantation period. Evidence-Based complementary Altern medicine: eCAM (2017) 2017:3647080. doi: 10.1155/2017/3647080 PMC5446881 28588637

[16] Bostancı MS Budak Ö Çakiroğlu H Gök K Köse O Çokluk E. The effect of protection of platelet-rich plasma against experimental ischemia/reperfusion injury in the rat ovary on *in vitro* fertilization outcomes. J obstetrics gynaecol Res (2022) 48:1390–8. doi: 10.1111/jog.15232 35322499

[17] Chen W Chen J Xu M Zhong Z Zhang Q Yang W. Electroacupuncture facilitates implantation by enhancing endometrial angiogenesis in a rat model of ovarian hyperstimulation. Biol Reprod (2019) 100:268–80. doi: 10.1093/biolre/ioy176 PMC6335210 30084973

[18] Gong X Lou J Lu Q Huang H Jin Z. Bu shen huo xue decoction restores endometrial leukemia-inhibitory factor but not angiopoietin-2 expression, and improves uterine receptivity in the controlled ovarian stimulation rat model. Exp Ther Med (2015) 9:751–7. doi: 10.3892/etm.2015.2193 PMC4316862 25667623

[19] Lessey BA Young SL. What exactly is endometrial receptivity? Fertility sterility (2019) 111:611–7. doi: 10.1016/j.fertnstert.2019.02.009 30929718

[20] Zhang S Lin H Kong S Wang S Wang H Wang H. Physiological and molecular determinants of embryo implantation. Mol aspects Med (2013) 34:939–80. doi: 10.1016/j.mam.2012.12.011 PMC4278353 23290997

[21] Lessey BA. Adhesion molecules and implantation. J Reprod Immunol (2002) 55:101–12. doi: 10.1016/s0165-0378(01)00139-5 12062825

[22] Craciunas L Gallos I Chu J Bourne T Quenby S Brosens JJ. Conventional and modern markers of endometrial receptivity: A systematic review and meta-analysis. Hum Reprod Update (2019) 25:202–23. doi: 10.1093/humupd/dmy044 30624659

[23] Shan L Zhou Y Peng S Wang X Shan Z Teng W. Implantation failure in rats with subclinical hypothyroidism is associated with lif/stat3 signaling. Endocr connections (2019) 8:718–27. doi: 10.1530/ec-19-0185 PMC6547307 31063977

[24] Mokhtar MH Giribabu N Salleh N. Testosterone decreases the number of implanting embryos, expression of pinopode and l-selectin ligand (meca-79) in the endometrium of early pregnant rats. Int J Environ Res Public Health (2020) 17:2293. doi: 10.3390/ijerph17072293 32235321 PMC7177729

[25] Xi J Cheng J Jin CC Liu JY Shen ZR Xia LJ. Electroacupuncture improves pregnancy outcomes in rats with thin endometrium by promoting the expression of pinopode-related molecules. BioMed Res Int (2021) 2021:6658321. doi: 10.1155/2021/6658321 33937407 PMC8062184

[26] Xing L Chen Y He Z He M Sun Y Xu J. Acupuncture improves endometrial angiogenesis by activating pi3k/akt pathway in a rat model with pcos. Evidence-Based complementary Altern medicine: eCAM (2022) 2022:1790041. doi: 10.1155/2022/1790041 PMC9433287 36062171

[27] Zhao Y Park S Bagchi MK Taylor RN Katzenellenbogen BS. The coregulator, repressor of estrogen receptor activity (rea), is a crucial regulator of the timing and magnitude of uterine decidualization. Endocrinology (2013) 154:1349–60. doi: 10.1210/en.2012-2026 PMC3578990 23392257

[28] Miller PB Parnell BA Bushnell G Tallman N Forstein DA Higdon HL. Endometrial receptivity defects during ivf cycles with and without letrozole. Hum Reprod (Oxford England) (2012) 27:881–8. doi: 10.1093/humrep/der452 PMC3279128 22246449

[29] Cullinan EB Abbondanzo SJ Anderson PS Pollard JW Lessey BA Stewart CL. Leukemia inhibitory factor (lif) and lif receptor expression in human endometrium suggests a potential autocrine/paracrine function in regulating embryo implantation. Proc Natl Acad Sci United States America (1996) 93:3115–20. doi: 10.1073/pnas.93.7.3115 PMC39771 8610178

[30] Wang J Wang K. New insights into chlamydia pathogenesis: Role of leukemia inhibitory factor. Front Cell infection Microbiol (2022) 12:1029178. doi: 10.3389/fcimb.2022.1029178 PMC9623337 36329823

[31] Massimiani M Lacconi V La Civita F Ticconi C Rago R Campagnolo L. Molecular signaling regulating endometrium-blastocyst crosstalk. Int J Mol Sci (2019) 21:23. doi: 10.3390/ijms21010023 31861484 PMC6981505

[32] Nicola NA Babon JJ. Leukemia inhibitory factor (lif). Cytokine Growth factor Rev (2015) 26:533–44. doi: 10.1016/j.cytogfr.2015.07.001 PMC4581962 26187859

[33] Arici A Engin O Attar E Olive DL. Modulation of leukemia inhibitory factor gene expression and protein biosynthesis in human endometrium. J Clin Endocrinol Metab (1995) 80:1908–15. doi: 10.1210/jcem.80.6.7775640 7775640

[34] Vogiagis D Marsh MM Fry RC Salamonsen LA. Leukaemia inhibitory factor in human endometrium throughout the menstrual cycle. J Endocrinol (1996) 148:95–102. doi: 10.1677/joe.0.1480095 8568476

[35] Patel B Elguero S Thakore S Dahoud W Bedaiwy M Mesiano S. Role of nuclear progesterone receptor isoforms in uterine pathophysiology. Hum Reprod Update (2015) 21:155–73. doi: 10.1093/humupd/dmu056 PMC4366574 25406186

[36] Gui J Xiong F Yang W Li J Huang G. Effects of acupuncture on lif and il-12 in rats of implantation failure. Am J Reprod Immunol (New York NY: 1989) (2012) 67:383–90. doi: 10.1111/j.1600-0897.2011.01097.x 22229306

[37] You F Du X Zhang T Wang Y Lv Y Zeng L. Electroacupuncture improves endometrial receptivity through mirna-223-3p-mediated regulation of leukemia inhibitory factor/signal transducer and activator of transcription 3 signaling pathway. Bioengineered (2022) 13:10298–312. doi: 10.1080/21655979.2022.2062524 PMC9161864 35435116

[38] Rizov M Andreeva P Dimova I. Molecular regulation and role of angiogenesis in reproduction. Taiwanese J obstetrics gynecol (2017) 56:127–32. doi: 10.1016/j.tjog.2016.06.019 28420494

[39] Patel BG Rudnicki M Yu J Shu Y Taylor RN. Progesterone resistance in endometriosis: Origins, consequences and interventions. Acta obstetricia gynecologica Scandinavica (2017) 96:623–32. doi: 10.1111/aogs.13156 28423456

[40] Fukui Y Hirota Y Matsuo M Gebril M Akaeda S Hiraoka T. Uterine receptivity, embryo attachment, and embryo invasion: Multistep processes in embryo implantation. Reprod Med Biol (2019) 18:234–40. doi: 10.1002/rmb2.12280 PMC6613011 31312101

[41] Achache H Revel A. Endometrial receptivity markers, the journey to successful embryo implantation. Hum Reprod update (2006) 12:731–46. doi: 10.1093/humupd/dml004 16982667

[42] Song Y Zhou F Tan X Liu X Ding J Zhang C. Bushen huoxue recipe attenuates early pregnancy loss *via* activating endometrial cox2-pge2 angiogenic signaling in mice. BMC complementary Med therapies (2021) 21:36. doi: 10.1186/s12906-021-03201-9 PMC7809844 33446182

