# Supplementary figures and images for: Effect of acupuncture on the opening time of implantation window and endometrial receptivity in controlled ovarian hyperstimulation rats during peri-implantation period
Source: Front Endocrinol (Lausanne). 2023 Mar 17;14:1110266. doi: 10.3389/fendo.2023.1110266 (PMC10064091; doi:10.3389/fendo.2023.1110266)

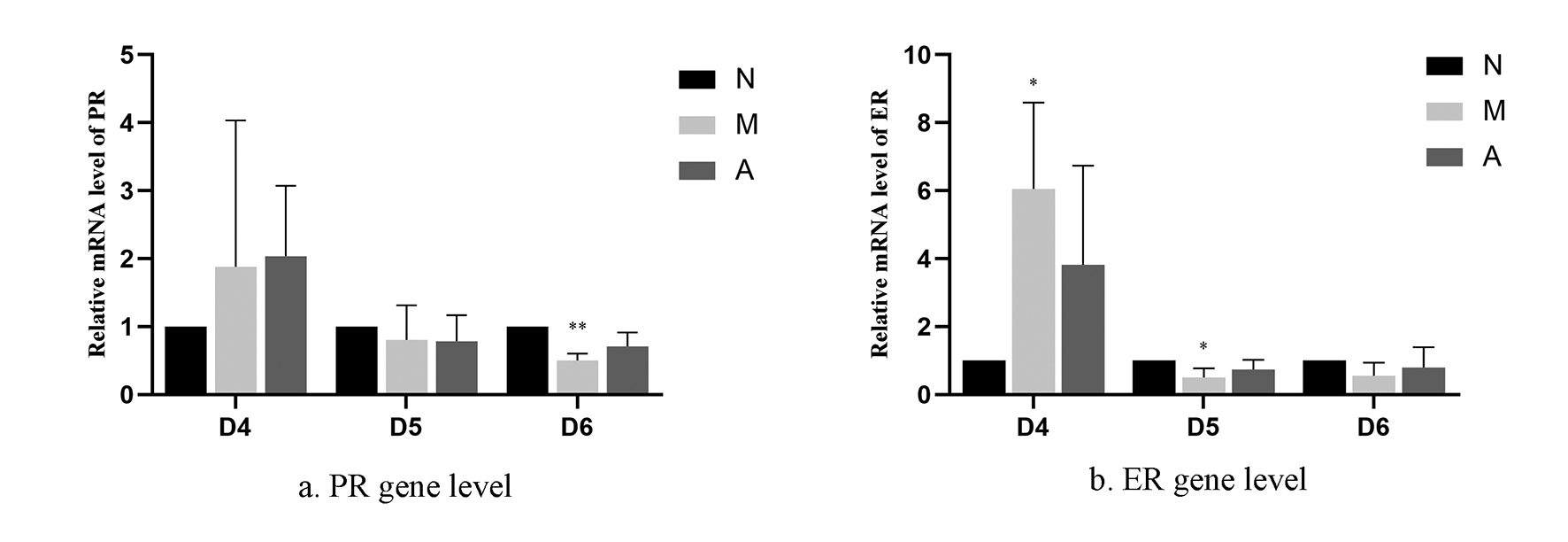

Supplement: Supplementary file 5 [file Image_1.jpeg]

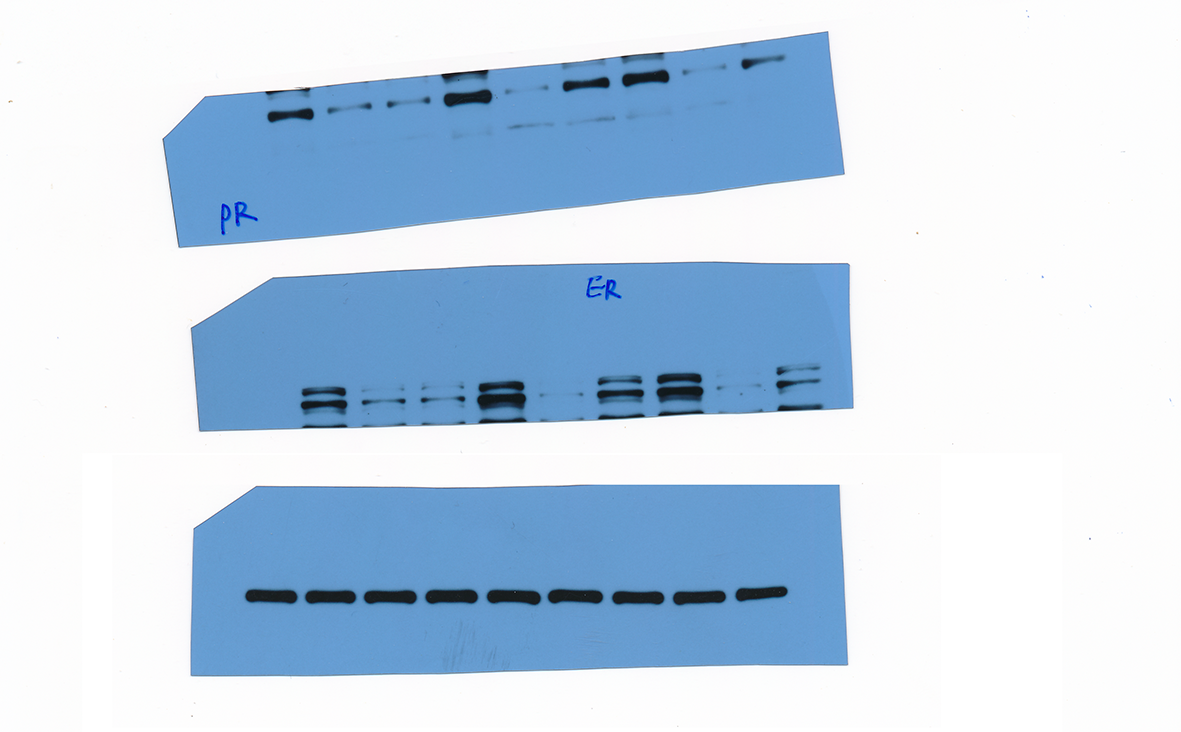

Supplement: Supplementary file 6 [file Image_2.tif]

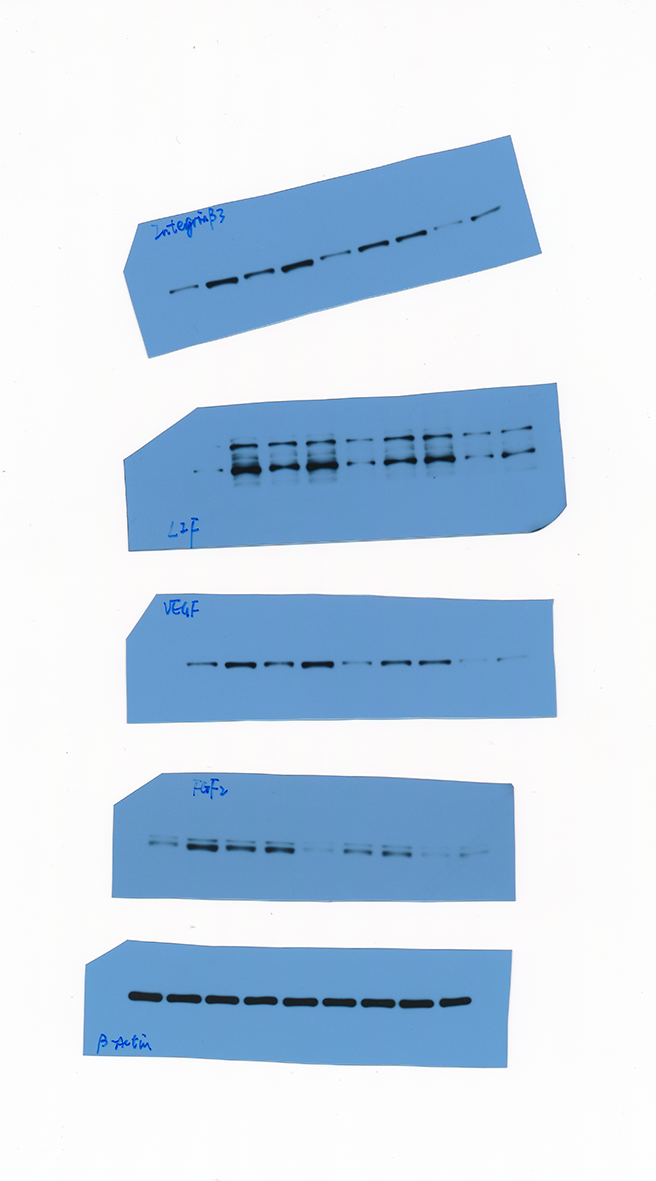

Supplement: Supplementary file 7 [file Image_3.tif]
